# Superior In Vitro Responses of a Native Rose Genotype to Driver Kuniyuki Walnut (DKW) Medium in a Comparative Study Using Natural and Synthetic Plant Growth Regulators

**DOI:** 10.3390/plants14162606

**Published:** 2025-08-21

**Authors:** Mahboubeh Davoudi Pahnekolayi, Zahra Parchianloo, Majid Babouyehdarabi, Meysam Ghasemi

**Affiliations:** 1 Department of Phytochemistry and Biochemistry of Natural Compounds, Institute of Chemical, Environmental and Bioscience Engineering, Technische Universität Wien (TU Wien), 1040 Vienna, Austria; 2 Department of Horticultural Sciences, Faculty of Agriculture, University of Zanjan, Zanjan M9PX+FX7, Iran; 3 Department of Plant and Environmental Sciences, Section for Crop Sciences, University of Copenhagen, Thorvaldsensvej 40, 1871 Frederiksberg C, Denmark

**Keywords:** calcium salts, culture media formulations, iron chelate, in vitro flowering, in vitro proliferation, potassium salts, silver nitrate

## Abstract

*Rosa canina* is one of the precious native rose rootstocks with a high reputation among plant producers, which has potential horticultural and pharmacological properties related to the cosmetic values and the production of secondary metabolites. Due to high horticultural consumption, applying the plant tissue culture technique as a major tool for healthy and massive-scale production of *R. canina* plants is not unexpected. However, the response of *R. canina* in vitro plantlets to various plant tissue culture ingredients is not well understood to tender an efficient applied protocol for qualitative and quantitative in vitro propagation. In this regard, the main objective of this study is to investigate the influence of several abiotic in vitro variants including six plant tissue culture media formulations (McCown’s Woody Plant Medium (WPM), Murashige and Skoog (MS), Van der Salm (VS), Schenk and Hildebrant (SH), Driver Kuniyuki Walnut (DKW), and Gamburg B5 (B5)) in combination with four concentrations (0, 1.5, 3, 4 mgL^−1^) of two types of cytokinins (6-Benzyaminopurine (BAP) and Kinetin (Kin)) simultaneously. Notably, it is perceived that DKW culture medium containing 1.5 mgL^−1^ BAP and 0.1 mgL^−1^ NAA is the best treatment for both in vitro morphological and flowering properties.

## 1. Introduction

Roses are the most prominent ornamental plants in the flower industry, which includes various economically valuable species. Following botanical taxonomy, they are divided into two groups: garden roses and cut roses. Garden roses, including native ones such as *Rosa canina* genotypes, are widespread, with high diversity in colors, cultivars, and climatic adaptations [1,2]. They are so precious for genetic engineering programs because they contain candidate gene pools for breeding [3]. Furthermore, they are used as a rootstock for grafting other garden and commercial rose cultivars to increase economic horticultural values such as cut flower yields [4]. Thus, establishing efficient propagation methods and maintaining *R. canina* genotypes as primary materials for breeding programs and as useful recognized rootstocks for grafting purposes are essential [2].

Regarding previous scientific achievements [5,6,7], the conventional propagation of native and commercial roses is highly limited by several factors, including their high heterozygous nature [8], polyploidy [9], and low and slow rates of propagation [10]. On the other hand, it is not economical to produce uniform healthy plants through traditional propagation methods by using a large number of mother plants [3,11]. Therefore, biotechnological methods, including in vitro propagation, are considered cost-effective for mass-scale, uniform, and healthy production of roses without time limitations [12].

As is known, not only from a propagation point of view, establishing biotechnological methods, including in vitro culture steps, is necessary in genetic engineering and genome editing programs for the insertion or modification of desired target genes via transformation skills. Thus, establishing an efficient in vitro protocol considering the high multiplication rate and plantlet quality of *R. canina* plays an important role in the successful biotechnological improvement of the species [13]. Several research studies on tissue culture of several species and cultivars of roses using different tissues with variable concentrations of Plant Growth Regulators (PGRs) and multiplication rates are available, but depending on genotypes and culture conditions, the efficiency is still low [1,2,3,5,6,7,14,15,16,17,18,19,20,21]. In addition to the importance of the multiplication rate of in vitro-produced plantlets, which has been the main focus of previous studies, the quality of the in vitro plantlets is also important. Since the plants produced in vitro will eventually enter the commercial market after the adaptation stage, maintaining good appearance quality is essential [14,15,19].

Previous studies demonstrated that several in vitro factors influence the propagation process to produce rose plantlets [5,12,19], including PGR types and concentrations [3,12,22], growth adjuvants [6,23,24,25], sucrose concentrations [26], photoperiod and lighting conditions quality [27]. Despite the importance of the mineral composition of culture medium on in vitro growth and morphogenic responses, there is no comprehensive report on studying various types of plant tissue culture media, including different ratios of macro- and microelements, on in vitro propagation of *R. canina*.

In vitro growth and morphogenic responses are highly influenced by the mineral composition of the culture medium [13]. Various plant tissue culture media have been introduced for the in vitro propagation of several plant species. Each nutrient in these culture media has its unique consequence on plant morphological, physiological, biochemical, and reproductive characteristics [28,29,30,31,32]. Insufficient or inappropriate amounts of the in vitro nutrients may disrupt normal plant metabolism, which results in a low propagation rate and poor appearance quality [30]. Murashige and Skoog basal medium (MS) [33] was initially developed for in vitro growth of tobacco calli and is the basal medium for many plant species. Over time, other plant culture media containing various nutrient compositions were introduced for in vitro methods, such as Lloyd and McCown’s Woody Plant Medium (WPM), Linsmaier and Skoog (LS), Van der Salm (VS), Schenk and Hildebrandt (SH), Driver Kuniyuki Walnut (DKW), Nitsch and Nitsch (N6), Gamborg B5 (B5), Whites medium (WH), etc. [11,21,29]. Differences in the nutrient contents of these culture media induce changes in in vitro growth parameters in plant species. For instance, among the various culture media, MS contains the highest levels of nitrogen (in both ammonium (NH_4_^+^) and nitrate (NO_3_^−^) forms), while WPM has the lowest nitrogen content. Similarly, SH is high in potassium (K) and DKW is high in calcium (Ca) and nitrate [30,31,32,33,34].

Although the MS medium is recognized as the basal culture medium for many plants, including rose genotypes, high nitrogen concentrations and imbalance ratios of nitrogen and ammonium are reported [34,35,36]. Moreover, in our previous studies [1,2], the MS medium was not suggested as an optimal medium for different native and commercial rose genotypes. To the best of our knowledge, no similar comprehensive studies were conducted on the response of various plant tissue culture media on in vitro morphological and reproductive characteristics of native *R. canina* genotypes. Therefore, the objective of this study is to determine an efficient in vitro nutrient medium for the native *R. canina* genotype by combining various concentrations of PGRs and plant tissue culture formulations by considering some in vitro organic additives.

## 2. Results

### 2.1. Significant Different Proliferation Rates of R. canina Explants in Response to Six In Vitro Culture Formulations Combined with PGRs

To investigate the prominent proliferation characteristics (proliferated shoot number, shoot height, shoot diameter, green leaves percentage) of *R. canina* explants, six various in vitro culture media in combination with four concentrations (0, 1.5, 3, 4 mgL^−1^) of two different cytokinins (BAP and Kin) were applied. The statistical analysis of the proliferated shoot number data demonstrated significant differences in the culture medium types (*F* (5, 150) = 46.66), cytokinin types (*F* (1, 150) = 212.855), cytokinin concentrations (*F* (3, 150) = 238.387), and their interactions (*F* (10, 150) = 5.621), (*p* ≤ 0.01). Following Table 1, the explants that had been treated with 1.5 mgL^−1^ BAP in the DKW medium displayed the highest proliferation capacity (*M* = 5.00, *SD* = 0.816). In contrast, the explants subcultured in control treatments for all six culture media (*M* = 1.00, *SD* = 0.00) recorded the lowest shoot number during the proliferation stage (Table 1).

As shown in Table 1, plants treated with BAP responded better than Kin in combination with 0.1 mgL^−1^ NAA. In addition to cytokinin types, the more effective concentration of cytokinin was related to 1.5 mgL^−1^, and the proliferation rate was obviously decreased by increasing the PGRs concentrations. Among six different culture media, DKW medium (*M* = 5.00, *SD* = 0.816) was the best one, followed by VS (*M* = 4.25, *SD* = 0.50) and MS (*M* = 2.75, *SD* = 0.50) media ingredients, which in combination with 1.5 mgL^−1^ BAP, had an impressive effect on proliferating capacity compared to others (Table 1), the lowest number of proliferated shoots was observed in the SH medium (*M* = 1.00, *SD* = 0.00). In a 1.5 mgL^−1^ concentration of BAP (the most effective concentration of cytokinin), WPM and B5 media did not demonstrate significant differences compared to each other.

Recording the length of in vitro proliferated shoots influenced by various treatments demonstrated significant differences in all variables including culture medium types (*F* (5, 150) = 640.428), cytokinin types (*F* (1, 150) = 75.189), cytokinin concentrations (*F* (2, 150) = 288.924), and their interactions (*F* (10, 150) = 4.229), (*p* ≤ 0.01). Without considering cytokinin types, increasing cytokinin concentrations induced stunted shoots in comparison with the control treatments in all six culture media (Table 1). Due to variable nutrient element ratios in different experimental culture media, various shoot lengths were observed. As seen in Table 1, the longest proliferated shoots were recorded in the DKW medium without any PGRs (*M* = 5.20, *SD* = 0.226). In addition to DKW medium, the longest shoots were revealed in VS and MS media, respectively (Table 1).

Following Table 1, the ANOVA analysis of the shoot diameter characteristic demonstrated significant differences between examined treatments including culture medium types (*F* (5, 150) = 6.468), cytokinin types (*F* (1, 150) = 21.930), cytokinin concentrations (*F* (2, 150) = 796.107), and their interactions (culture medium × cytokinin type × cytokinin concentration) (*F* (10, 150) = 2.489) (*p* ≤ 0.01). The thickest shoots were observed in the control treatment of DKW medium without any PGRs (*M* = 0.478 cm, *SD* = 0.008), and the thinnest was related to the B5 medium including 4 mgL^−1^ BAP/Kin (*M* = 0.062, *SD* = 0.005) (Table 1).

The quality of in vitro leaves is an important factor for ensuring vigorous growth and high flower yield in the acclimatization stage [32]. As the growth and development of in vitro plantlets depend on several factors, which were explained comprehensively before, in this study, the percentage of in vitro-produced green leaves has been recorded in six different culture media due to their varied concentrations of macro- and microelements and their ratios. Following the results, all variable factors including culture medium types (*F* (5, 150) = 5.473), cytokinin types (*F* (1, 150) = 59.943), and cytokinin concentrations (*F* (2, 150) = 154.175) demonstrated significant differences, while the interactions between all variables (culture medium × cytokinin type × cytokinin concentration) did not indicate clear differences (*F* (10, 150) = 1.216) (*p* ≤ 0.01).

As shown in Table 2, the highest percentage of green leaves is recorded in the VS culture medium (*M* = 100%, *SD* = 0.00), and the highest percentage of green leaves was observed in the SH medium (*M* = 27.62%, *SD* = 2.80). Among different treatments of cytokinin types and concentrations, the best results were related to the control treatments (*M* = 80.79%, *SD* = 24.11).

### 2.2. Suitable Combinations of Culture Media Formulations, PGR Types, and Concentrations can Induce Qualified In Vitro R. canina Flowers

In this study, an investigation of various culture medium ingredients in combination with varied types and concentrations of PGRs was applied. Analyzing experimental treatments declared that all the flowering characteristics, including flower number, flower stem number, flower diameter, flower stem length, and flower stem diameter, were statistically significant (*p* ≤ 0.01). Analyzing the flower number data demonstrated a significant influence of culture medium types (*F* (5, 150) = 86.826), cytokinin types (*F* (1, 150) = 100.082), cytokinin concentrations (*F* (2, 150) = 178.023), and their interactions (culture medium × cytokinin type × cytokinin concentration) (*F* (10, 150) = 3.709) (*p* ≤ 0.01). Flowering induction happened in DKW, MS, and VS culture media. No flower was detected in WPM, SH, and B5 media. As shown in Table 3, the highest number of in vitro flowers (3.50 ± 0.577) was recorded in 1.5 mgL^−1^ BAP, but there was a significant difference between the VS and DKW culture media, and they both demonstrated the highest flower numbers. Among various types and concentrations of PGRs, 1.5 mgL^−1^ BAP was the best treatment for rose in vitro flowering.

The effects of culture medium types (*F* (5, 150) = 71.725), cytokinin types (*F* (1, 150) = 59.559), cytokinin concentrations (*F* (2, 150) = 125.735), and their interactions (culture medium × cytokinin type × cytokinin concentration) (*F* (10, 150) = 3.578) on flower stem number were glaringly clear (*p* ≤ 0.01). As above, the highest number of flower stems (3.50 ± 0.577) was recorded in the 1.5 mgL^−1^ BAP treatment (Table 3). Our results demonstrated that every induced flower stem produced one flower, and it is obvious that by increasing the number of flower stems in roses, the number of flowers will also increase. One of the outstanding results of this study was that inducing flower stems did not occur in SH, WPM, and B5 media, which supports the importance of culture medium ingredients as one effective factors for in vitro flowering. In addition to culture medium formulation, the PGR type and concentration, and their interactions with each other, will finally determine the flowering induction capacity.

**Figure 1:**
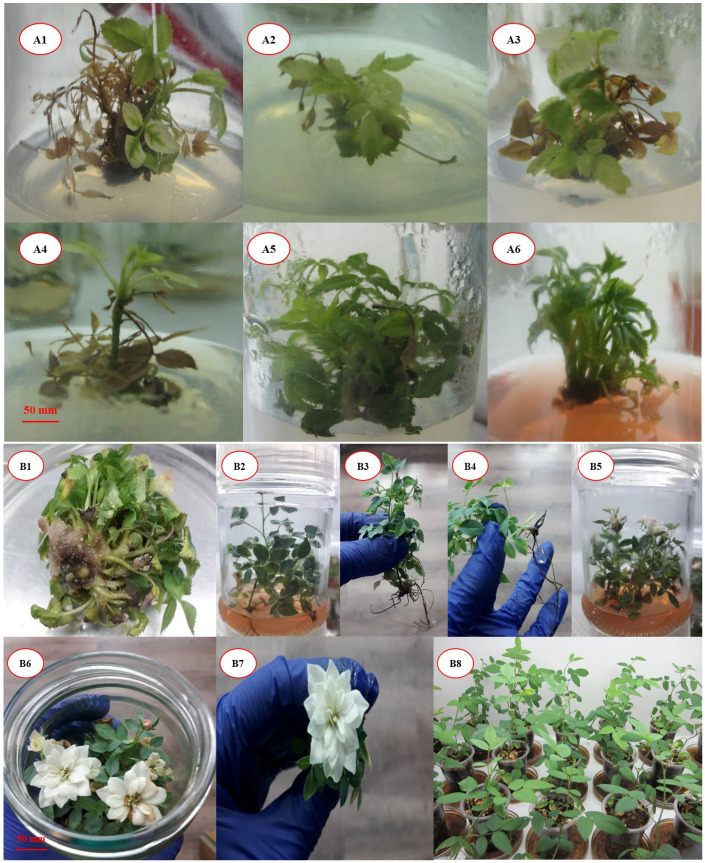
Morphological and flowering characteristics of *R. canina* plantlets under various culture media formulations: (**A1**–**A6**) quality of the regenerated shoots in different culture media: MS, B5, WPM, SH, DKW, VS. (**B1**) The highest multiplication rate in DKW medium containing 1.5 mgL^−1^ BAP + 0.1 mgL^−1^ NAA. (**B2**) The best quality of proliferated shoots in VS medium. (**B3**, **B4**) The best quality of in vitro produced plantlet by changing the culture medium ingredients. (**B5**) Flowering stems produced in VS medium containing 1.5 mgL^−1^ BAP. (**B6**) The highest flower diameter produced in DKW medium. (**B7**) The quality of a single flower induced in DKW medium. (**B8**) the quality of successfully acclimated plants.

Similar to other flowering characteristics, the effects of culture medium types (*F* (5, 150) = 207.495), cytokinin types (*F* (1, 150) = 278.852), cytokinin concentrations (*F* (2, 150) = 264.998), and their interactions (culture medium × cytokinin type × cytokinin concentration) (*F* (10, 150) = 7.668) on flower stem length demonstrated significant differences (*p* ≤ 0.01). A noteworthy result of this study is the difference in PGR functions in various developmental stages of plants in vitro. The results of both multiplication and flowering stages demonstrated that 1.5 mgL^−1^ BAP is an efficient treatment for *R. canina* in vitro propagation. In the multiplication stage, the best proliferation treatment did not result in the highest shoot length, while in the flowering stage, the best flowering treatment also resulted in the highest flower stem length. Our results prove that the interaction of PGRs and in vitro medium ingredients induces different responses in different plant developmental stages (Figure 1).

As it is described above, the significant differences in the effects of culture medium types (*F* (5, 150) = 184.388), cytokinin types (*F* (1, 150) = 17.707), cytokinin concentrations (*F* (2, 150) = 146.278), and their interactions (culture medium × cytokinin type × cytokinin concentration) (*F* (10, 150) = 3.279) on flower stem diameter were not unexpected (*p* ≤ 0.01). According to Table 3, the thickest flowers were formed in DKW medium containing 1.5 mgL^−1^ BAP or Kin. As it is demonstrated in Table 3, there were no significant differences between the same concentrations of BAP and Kin (1.5 mgL^−1^), but BAP resulted in a slightly larger flower stem diameter than Kin. Among DKW, MS, and VS culture media in which the flowering induction occurred, the lowest flowering characteristics were observed in high and low concentrations of PGRs in the MS medium.

The flower diameter was significantly influenced by various culture medium types (*F* (5, 150) = 233.727), cytokinin types (*F* (1, 150) = 166.949), cytokinin concentrations (*F* (2, 150) = 269.079), and their interactions (culture medium × cytokinin type × cytokinin concentration) (*F* (10, 150) = 12.625) (*p* ≤ 0.01). Flower diameter is one of the most prominent characteristics determining flower quality and is an important trait for in vitro rose breeders. Our results indicate that rose in vitro plantlets subcultured in DKW medium containing 1.5 mgL^−1^ BAP produced the thickest flowers.

## 3. Discussion

The VS medium was suggested as the optimum culture medium for rose tissue culture with different targets, which was comprehensively explained in our previous study [2]. This study has so far focused on the impact of PGRs, basal salt components of six different culture media, and their interactions to provide a comprehensive, efficient protocol for in vitro culture of roses.

An important macroelement for in vitro shoot multiplication and elongation is potassium (K). It is one of the main components implicated in the biochemical, structural, catalytic, and electrochemical functions of higher plants. It plays a crucial role in regulating various physiological processes that influence in vitro morphological and flowering characteristics via plant cell membrane ion homeostasis, enhancement of nutrient uptake, cell division and mitosis, and the formation of cell walls [37]. On the other hand, it is one of the outstanding macroelements that can deal with the Reactive Oxygen Species (ROS) production and activity due to in vitro stress conditions (light, temperature, and wounding practices) that reduce oxidative stress [38,39]. Potassium exists in different forms and ratios in these six studied culture media (Appendix A) and is provided by the KNO_3_, K_2_SO_4_, and K_2_H_2_PO_4_ salts. Regarding our previous study [2], suggesting that VS medium was more efficient than MS medium for *R. canina* in vitro culture, our current results in this study confirmed this truth again. The DKW medium responds better than the VS medium, which is absolutely related to the macro and micro ratios and their interactions with other in vitro ingredients. DKW medium is the only culture medium that contains the maximum levels of K_2_SO_4_ and K_2_H_2_PO_4_. However, it does not contain any KNO_3_. The results of this study showed that the combination of K_2_SO_4_ and K_2_H_2_PO_4_ in the DKW medium responded better for improving physiological characteristics than the combination of KNO_3_ and K_2_H_2_PO_4_ in MS and VS media. Moreover, the prominent role of K_2_SO_4_ in combination with MgSO_4_ on the direct and indirect regeneration pathways and embryo development in somatic embryogenesis was reported before [37].

On the other hand, Plant Tissue Culture Media include varied concentrations of calcium (Ca) salts. Various Ca salts in culture media have a strong effect on biomass growth, cell wall organization, somatic embryogenesis, and plant regeneration productivity [35]. It is confirmed that Ca, alone or in combination with other organic materials, has a prominent role in some metabolic pathways [37,38,39]. According to Appendix A; MS, VS, B5, and SH media consist of 440, 440, 150, and 200 mgL^−1^ CaCl_2_H_2_O, respectively. Both forms of calcium (Ca), including calcium chloride (CaCl_2_) and calcium nitrate (CaNO_3_) were found in DKW and WPM media. In all treatments, there was a greater increase in shoot diameter measurement in the explants grown in media containing both types of chloride and nitrate of Ca, in which the DKW medium provided the highest shoot diameter due to high concentrations of Ca salts. Pasternak and Steinmacher [38] confirmed that the enhancement of calcium chloride in tissue culture media can possibly increase Ca content in the shoots, which leads to effective shoot regeneration. Moreover, the combination of Ca salts with other in vitro elements, especially boron (B), can decrease the vitrification possibility. Regarding Table 1, the thinnest shoots were observed in the B5 medium, which contains the lowest concentration of Ca (150 mgL^−1^).

In vitro leaf chlorosis and necrosis are mostly indicative of iron deficiency [36]. Leaf chlorosis did not occur in the VS medium containing FEEDDHA instead of FEEDTA. The stability of chelated iron in the culture medium is essential for its availability under in vitro conditions, which requires a pH range of 5 to 6. However, under light conditions and at a pH of 5.7, the FEEDTA oxidizes to insoluble ferric oxide, leading to immediate iron deficiency, and indicating leaf chlorosis. Due to the photostability of FEEDDHA over FEEDTA, iron is more readily available to rose in vitro plantlets, resulting in enhanced green leaf percentage and leaf quality [40,41,42,43,44]. Zawadzka & Orlikowska [45] showed that FEEDDHA increases the quality and multiplication rate of raspberry cultures by eliminating leaf chlorosis and necrosis in three cultivars “Canby”, “Beskid”, and “Veten”. Furthermore, the impact of two different MS media containing FEEDTA and FEEDDHA on the multiplication and growth rate of five cultivars of gerbera was tested, and it was found that leaf yellowing occurred in the FEEDTA-containing medium. In contrast, leaf greening was observed in medium containing FEEDDHA. Our results highlight the substantial influence of medium type, specifically the presence of FEEDTA or FEEDDHA, on in vitro rose growth responses. Our previous research results demonstrated that the proliferated shoots in the VS medium containing FEEDDHA were fresher and more developed for subculture than the shoots in other culture media containing FEEDTA, emphasizing the critical role of iron as an essential element for in vitro rose shoot development.

Among treatments involving different cytokinin types and concentrations, the best results were observed in the control treatments, indicating the fact that the quality of in vitro plantlets depends more on culture medium ingredients (macroelements, microelements, and vitamins) and culture conditions (light intensity, daylight, and temperature) than on PGR types and concentrations. In addition to the in vitro culture medium type, the interaction between endogenous hormone amount and the exogenous application of PGRs in vitro situation determines the final morphological and flowering development of in vitro explants [41]. Among different cytokinins used for in vitro cultivation of rose genotypes, BAP is the most effective one, especially for shoot multiplication. For example, previous research studies on in vitro propagation of *R. canina* and *R. hybrida* demonstrated higher shoot number, shoot length, and number of leaves in 1–2 mgL^−1^ BAP along with a low concentration of NAA (0.1 mgL^−1^) [44,45,46,47]. Usually, the optimum concentration of PGRs influencing morphological and flowering performances of in vitro production is not only species-specific, but also varies in combination with other internal (explant type, genotype, cultivar, and endogenous hormones) and external (environmental factors and endogenous application of PGRs) factors [48]. Interestingly, our findings highlighted the undeniable role of the in vitro culture medium on the qualitative and quantitative in vitro production of *R. canina.*

In vitro flowering is an important target for rose breeders in in vitro breeding and genetic engineering programs. During the floral transition that subsequently terminates after generating the floral meristem and floral organs, the level of endogenous hormones will change. This alteration consists of cytokinin and gibberellin enhancement and cytokinin/auxin ratio reduction that leads to flower formation [41]. In total, various in vitro factors, including plant tissue culture media formulations, PGR types and concentrations, sucrose concentration, and environmental conditions, determine in vitro flowering. Regarding the flowering function that occurred in MS, VS, and DKW media in our study, the higher nitrate level in DKW medium compared to ammonium possibly supports floral induction. Nitrogen status is an important in vitro component that can regulate flowering gene expression in plants [49]. In control treatments for these three media, no flowering induction was recorded, while in other treatments with various PGR concentrations and types, the flowering was induced in different ranges. It shows that, in addition to the importance of the culture medium formulations on the quality of rose in vitro flowers, the type and concentration of PGRs have a significant influence on flowering induction. The effect of PGRs and growth adjuvants on rose in vitro flowering induction depends highly on the genotypic diversity and explant age. The initial explants should be selected from mature mother plants, and depending on the rose cultivar, flowering will occur with the use of PGRs and growth adjuvants in combination with the plant culture media formulation [50]. According to Kang et al. [42], depending on plant species, in vitro flowers will be induced in different nutritional induction media. On the other hand, Hong et al. [50] reported that the total time from the initial culture of the explant and the subculture time before the flowering are the two main factors for in vitro flowering induction. Previous studies have proven that due to the various levels of phytohormones, growth adjuvants (especially internal proline), and their interaction with other in vitro culture medium ingredients during the multiplication stage, the physiological status of the transferred shoots to the flowering stage will be different [51,52,53,54].

In this study, Kin didn’t have an eminent effect on flowering induction, while Ali and Mangrio [54] reported the production of *Rosa* sp. floral bud in MS + 1.5 ppm Kin + 2.5 ppm spermidine + 0.1 ppm NAA. Bimal et al. [55] reported the highest flowering induction in the medium containing BAP, coconut water, and 2-4-D, the same as the highest proliferation results. Similar to Bimal et al. [55], in our study, the best treatment (1.5 mgL^−1^ BAP in DKW medium) in the proliferation stage also demonstrated the highest flowering induction.

## 4. Materials and Methods

### 4.1. Plant Material Collection, Sterilization, and Initial Establishment

The one-year-old healthy alternative branches of the *R. canina* genotype of the Shahmirzad area of Semnan province, Iran (35°46′22″ N, 53°19′43″ E), were collected in summer and transferred to the plant tissue culture laboratory. Leafless nodal segments with approximately 1–1.5 cm length were selected for sterilization and initial establishment. Both sterilization and initial establishment phases were carried out according to [2], with some minor modifications. For the sterilization stage, all nodal segments were washed with running tap water for 1 h, and their surface disinfections were performed with 1% Carbendazim fungicide for 15 min, 70% (*w*/*v*) Ethanol for 60 s, and 2.5% (*w*/*v*) Sodium hypochlorite for 15 min. Eventually, they were washed three times with double-distilled water with intervals of 5, 10, and 15 min. MS basal medium supplemented with 100 mgL^−1^ Silver nitrate (AgNO_3_) and 200 mgL^−1^ Cefotaxime antibiotic was used as the establishment culture medium.

### 4.2. Six Various Culture Media Formulations Were Prepared for the In Vitro Proliferation Stage

For in vitro proliferation of axillary shoots from nodal stem segments, a full factorial experiment in a Completely Randomized Design (CRD) with 48 treatments and 5 repetitions was designed. Six various formulations of plant tissue culture media (Lioyd and McCown (WPM), Murashige and Skoog (MS), Van der Salm (VS), Schenk and Hildebrandt (SH), Driver Kuniyuki Walnut (DKW), and Gamborg B5 (B5)) (Appendix A) in combination with four concentrations (0, 1.5, 3, 4 mg L^−1^) of two types of cytokinins (6-Benzylaminopurine (BAP) and Kinetin (Kin)) were prepared for the proliferation stage. A stable concentration of 0.1 mgL^−1^ α-Naphthaleneacetic acid (NAA) (0.1 mgL^−1^) was added to all cytokinin treatments.

### 4.3. In Vitro Rooting and Ex Vitro Plant Acclimatization

After the proliferation stage, all suitable in vitro shoots (1–1.5 cm length) were transferred to the best medium, determined in the proliferation stage, for the rooting stage. PGR types and concentrations in the rooting stage were applied according to our previous studies [1,2]: ½ DKW + 0.3 mgL^−1^ Indole-3-butyric acid (IBA) + 0.3 mgL^−1^ NAA. The rooting process was prolonged for 6 to 8 weeks. For the first acclimatization process, transparent plastic bags were applied to avoid losing extra relative humidity. The first acclimatization phase was carried out in the tissue culture room with a temperature of 25 ± 1 °C and 80% relative humidity for one month. Finally, the acclimatized plantlets were transferred to the greenhouse with a temperature of 25 ± 1 °C and 60% relative humidity after the successful first acclimatization stage. In total, 98% of the plantlets were acclimatized successfully.

### 4.4. In Vitro Cultures and Growth Conditions

All in vitro cultures were carried out in glass bottle jars containing at least 30–35 mL of culture medium (following the treatments). Adjusting the pH of all culture media to 5.7–5.85 was necessary before agar addition and autoclaving. Autoclaving at 121 °C for 20 min was applied for sterilizing all in vitro equipment and media before cultures. For all in vitro culture steps, including the establishment, proliferation, and rooting stages, the in vitro jars were maintained at a temperature of 23 ± 1 °C and 16/8 (light/dark) photoperiods with T-10 fluorescent lamps at 37.5 µmol m^−2^ s^−1^ photosynthetic photon flux (PPF) in the incubation room. All culture medium macro and micro elements, vitamins, agar, and sucrose were selected from Sigma-Aldrich (St. Louis, MO, USA), and PGRs were purchased from the Duchefa company (Haarlem, The Netherlands).

### 4.5. Experimental Design and Statistical Analysis

Full factorial experiments in CRD with specified treatments and repetitions were carried out for all investigations. For data collection and analysis, the three-way analysis of variance (three-way ANOVA, SPSS version 28.0) was applied carefully. The statistical clearance was determined at a 95% confidence level, and the LSD post hoc test (*p* ≤ 0.05) was used for the separation of mean values. In the proliferation stage, in vitro morphological and flowering data included proliferated shoot number, proliferated shoot height (cm), shoot diameter (cm), green leaf percentage (%) (healthy vs. chlorotic leaves), flower number, floral stick number, floral stick length (cm), flower diameter (cm), and floral stick diameter (cm) were applied after 8 weeks. Shoot diameter and floral stick diameter were measured from the basal parts of the stem in the proliferation and flowering stages, respectively.

## Figures and Tables

**Table 1:** Morphological variables of *R. canina* explants proliferated in vitro in various plant culture media combined with different types and concentrations of PGRs.

Treatments	Proliferated No. of Shoots	Shoot Height (cm)	Shoot Diameter (cm)
WPM + Control	1.00 ± 0.00 f	1.30 ± 0.329 ghij	0.307 ± 0.004 e
WPM + 1.5 mgL−1 BAP	2.25 ± 0.50 d	1.32 ± 0.125 fghi	0.282 ± 0.005 f
WPM + 3 mgL−1 BAP	1.00 ± 0.00 f	0.85 ± 0.129 kl	0.257 ± 0.005 g
WPM + 4 mgL−1 BAP	1.00 ± 0.00 f	0.42 ± 0.050 mn	0.232 ± 0.005 h
WPM + 1.5 mgL−1 Kin	1.25 ± 0.50 ef	1.45 ± 0.129 fgh	0.280 ± 0.00 f
WPM + 3 mgL−1 Kin	1.00 ± 0.00 f	0.60 ± 0.141 lmn	0.255 ± 0.005 g
WPM + 4 mgL−1 Kin	1.00 ± 0.00 f	0.325 ± 0.095 no	0.230 ± 0.00 h
DKW + Control	1.00 ± 0.00 f	5.20 ± 0.226 a	0.487 ± 0.008 a
DKW + 1.5 mgL−1 BAP	5.00 ± 0.816 a	3.15 ± 0.129 c	0.442 ± 0.005 b
DKW + 3 mgL−1 BAP	2.25 ± 0.50 d	2.15 ± 0.132 e	0.352 ± 0.012 d
DKW + 4 mgL−1 BAP	1.50 ± 0.57 e	1.45 ± 0.310 fgh	0.312 ± 0.005 e
DKW + 1.5 mgL−1 Kin	2.00 ± 0.00 d	2.57 ± 0.095 d	0.430 ± 0.00 c
DKW + 3 mgL−1 Kin	1.00 ± 0.00 f	1.07 ± 0.095 ijk	0.347 ± 0.005 d
DKW + 4 mgL−1 Kin	1.00 ± 0.00 f	1.05 ± 0.057 ijk	0.307 ± 0.005 e
MS + Control	1.00 ± 0.00 f	3.07 ± 0.088 c	0.265 ± 0.011 g
MS + 1.5 mgL−1 BAP	2.75 ± 0.50 c	1.95 ± 0.57 e	0.208 ± 0.008 i
MS + 3 mgL−1 BAP	1.50 ± 0.57 e	1.37 ± 0.43 fgh	0.185 ± 0.005 jk
MS + 4 mgL−1 BAP	1.25 ± 0.50 ef	1.00 ± 0.00 jk	0.162 ± 0.005 mn
MS + 1.5 mgL−1 Kin	2.00 ± 0.00 d	1.52 ± 0.309 fg	0.207 ± 0.005 i
MS + 3 mgL−1 Kin	1.00 ± 0.00 f	1.00 ± 0.00 jk	0.180 ± 0.00 jk
MS + 4 mgL−1 Kin	1.00 ± 0.00 f	1.00 ± 0.00 jk	0.160 ± 0.00 mn
VS + Control	1.00 ± 0.00 f	4.07 ± 0.088 b	0.265 ± 0.011 g
VS + 1.5 mgL−1 BAP	4.25 ± 0.50 b	2.77 ± 0.320 d	0.210 ± 0.008 i
VS + 3 mgL−1 BAP	2.25 ± 0.50 d	1.62 ± 0.434 f	0.185 ± 0.005 jk
VS + 4 mgL−1 BAP	1.50 ± 0.57 e	1.00 ± 0.00 jk	0.162 ± 0.005 mn
VS + 1.5 mgL−1 Kin	2.00 ± 0.00 d	1.62 ± 0.150 f	0.207 ± 0.005 i
VS + 3 mgL−1 Kin	1.00 ± 0.00 f	1.00 ± 0.00 jk	0.180 ± 0.00 jk
VS + 4 mgL−1 Kin	1.00 ± 0.00 f	1.00 ± 0.00 jk	0.160 ± 0.00 mn
SH + Control	1.00 ± 0.00 f	0.67 ± 0.046 lm	0.205 ± 0.011 i
SH + 1.5 mgL−1 BAP	1.00 ± 0.00 f	0.45 ± 0.57 mn	0.187 ± 0.005 j
SH + 3 mgL−1 BAP	1.00 ± 0.00 f	0.125 ± 0.050 o	0.175 ± 0.005 kl
SH + 4 mgl−1 BAP	1.00 ± 0.00 f	0.125 ± 0.050 o	0.152 ± 0.009 mn
SH + 1.5 mgL−1 Kin	1.00 ± 0.00 f	0.45 ± 0.057 mn	0.182 ± 0.005 jk
SH + 3 mgL−1 Kin	1.00 ± 0.00 f	0.125 ± 0.050 o	0.167 ± 0.005 lm
SH + 4 mgL−1 Kin	1.00 ± 0.00 f	0.125 ± 0.050 o	0.115 ± 0.010 o
B5 + Control	1.00 ± 0.00 f	2.07 ± 0.088 e	0.157 ± 0.008 mn
B5 + 1.5 mgL−1 BAP	2.25 ± 0.50 d	1.45 ± 0.251 fgh	0.087 ± 0.005 p
B5 + 3 mgL−1 BAP	1.00 ± 0.00 f	1.17 ± 0.206 hij	0.077 ± 0.005 p
B5 + 4 mgL−1 BAP	1.00 ± 0.00 f	1.00 ± 0.00 jk	0.062 ± 0.005 q
B5 + 1.5 mgL−1 Kin	1.25 ± 0.50 ef	1.52 ± 0.309 fg	0.087 ± 0.005 p
B5 + 3 mgL−1 Kin	1.00 ± 0.00 f	1.00 ± 0.00 jk	0.077 ± 0.005 p
B5 + 4 mgL−1 Kin	1.00 ± 0.00 f	1.00 ± 0.00 jk	0.62 ±0.005 q

The same lowercase letters indicate no significant differences between treatments according to the LSD test at a 5% probability level (mean ± standard deviation).

**Table 2:** The percentage (%) of green leaves of *R. canina* explants proliferated in vitro in various plant culture media, including different types and concentrations of PGRs.

Source of Variants	Green Leaf Percentage (%)
**Plant Culture Media**	
WPM	63.21 ± 7.05 d
DKW	97.25 ± 3.38 b
MS	79.31 ± 8.58 c
VS	100.00 ± 0.00 a
SH	27.62 ± 2.80 e
B5	73.31 ± 5.85 c
**Cytokinin type**	
Control	80.79 ± 24.11 a
BAP	73.65 ± 25.54 b
Kin	71.02 ± 24.74 c
**Cytokinin concentration (mgL^−1^)**	
0	80.79 ± 24.11 a
1.5	75.97 ± 25.05 b
3	72.35 ± 24.90 c
4	68.68 ± 25.31 d

The same lowercase letters indicate no significant differences between various treatments according to the LSD test at a 5% probability level (mean ± standard deviation).

**Table 3:** Flowering variables of *R. canina* explants in vitro in various plant culture media combined with different types and concentrations of PGRs.

Treatments	Flower Number	Flower Stem Number	Flower Stem Length (cm)	Flower Stem Diameter (cm)	Flower Diameter (cm)
WPM + Control	0.00 ± 0.00 e	0.00 ± 0.00 f	0.00 ± 0.00 i	0.00 ± 0.00 e	0.00 ± 0.00 i
WPM + 1.5 mgL^−1^ BAP	0.00 ± 0.00 e	0.00 ± 0.00 f	0.00 ± 0.00 i	0.00 ± 0.00 e	0.00 ± 0.00 i
WPM + 3 mgL^−1^ BAP	0.00 ± 0.00 e	0.00 ± 0.00 f	0.00 ± 0.00 i	0.00 ± 0.00 e	0.00 ± 0.00 i
WPM + 4 mgL^−1^ BAP	0.00 ± 0.00 e	0.00 ± 0.00 f	0.00 ± 0.00 i	0.00 ± 0.00 e	0.00 ± 0.00 i
WPM + 1.5 mgL^−1^ Kin	0.00 ± 0.00 e	0.00 ± 0.00 f	0.00 ± 0.00 i	0.00 ± 0.00 e	0.00 ± 0.00 i
WPM + 3 mgL^−1^ Kin	0.00 ± 0.00 e	0.00 ± 0.00 f	0.00 ± 0.00 i	0.00 ± 0.00 e	0.00 ± 0.00 i
WPM + 4 mgL^−1^ Kin	0.00 ± 0.00 e	0.00 ± 0.00 f	0.00 ± 0.00 i	0.00 ± 0.00 e	0.00 ± 0.00 i
DKW + Control	0.00 ± 0.00 e	0.00 ± 0.00 f	0.00 ± 0.00 i	0.00 ± 0.00 e	0.00 ± 0.00 i
DKW + 1.5 mgL^−1^ BAP	3.50 ± 0.577 a	3.50 ± 0.577 a	1.80 ± 0.244 a	0.442 ± 0.005 a	0.975 ± 0.095 a
DKW + 3 mgL^−1^ BAP	1.50 ± 0.577 c	1.50 ± 0.577 c	0.97 ± 0.125 b	0.352 ± 0.012 b	0.450 ± 0.057 cd
DKW + 4 mgL^−1^ BAP	0.50 ± 0.577 d	0.50 ± 0.577 e	0.225 ± 0.263 fg	0.157 ± 0.181 c	0.175 ± 0.206 f
DKW + 1.5 mgL^−1^ Kin	1.50 ± 0.577 c	1.50 ± 0.577 c	0.875 ± 0.095 bc	0.430 ± 0.00 a	0.450 ± 0.057 cd
DKW + 3 mgL^−1^ Kin	0.50 ± 0.577 d	0.50 ± 0.577 e	0.20 ± 0.230 gh	0.347 ± 0.005 b	0.325 ± 0.050 e
DKW + 4 mgL^−1^ Kin	0.00 ± 0.00 e	0.00 ± 0.00 f	0.00 ± 0.00 i	0.00 ± 0.00 i	0.00 ± 0.00 i
MS + Control	0.00 ± 0.00 e	0.00 ± 0.00 f	0.00 ± 0.00 i	0.00 ± 0.00 i	0.00 ± 0.00 i
MS + 1.5 mgL^−1^ BAP	2.75 ± 0.500 b	2.50 ± 0.577 b	0.80 ± 0.081 c	0.210 ± 0.008 c	0.675 ± 0.095 b
MS + 3 mgL^−1^ BAP	1.00 ± 0.00 e	1.00 ± 0.00 d	0.40 ± 0.081 e	0.185 ± 0.005 c	0.500 ± 0.00 c
MS + 4 mgL^−1^ BAP	0.00 ± 0.00 e	0.00 ± 0.00 f	0.00 ± 0.00 i	0.0 ± 0.0 e	0.00 ± 0.00 i
MS + 1.5 mgL^−1^ Kin	1.50 ± 0.577 c	1.50 ± 0.577 c	0.325 ± 0.050 ef	0.207 ± 0.005 c	0.400 ± 0.081 d
MS + 3 mgL^−1^ Kin	0.50 ± 0.577 d	0.50 ± 0.577 e	0.100 ± 0.115 hi	0.090 ± 0.103 d	0.100 ± 0.115 gh
MS + 4 mgL^−1^ Kin	0.00 ± 0.00 e	0.00 ± 0.00 f	0.00 ± 0.00 i	0.00 ± 0.00 i	0.00 ± 0.00 i
VS + Control	0.00 ± 0.00 e	0.00 ± 0.00 f	0.00 ± 0.00 i	0.00 ± 0.00 i	0.00 ± 0.00 i
VS + 1.5 mgL^−1^ BAP	3.50 ± 0.577 a	1.75 ± 0.500 c	0.900 ± 0.081 bc	0.210 ± 0.008 c	0.275 ± 0.095 e
VS + 3 mgL^−1^ BAP	1.50 ± 0.577 c	1.50 ± 0.577 c	0.675 ± 0.050 d	0.185 ± 0.005 c	0.175 ± 0.050 f
VS + 4 mgL^−1^ BAP	0.50 ± 0.577 d	0.50 ± 0.577 e	0.225 ± 0.263 fg	0.082 ± 0.095 d	0.050 ± 0.057 hi
VS + 1.5 mgL^−1^ Kin	1.50 ± 0.577 c	1.50 ± 0.577 c	0.325 ± 0.050 ef	0.207 ± 0.005 c	0.125 ± 0.050 fg
**VS + 3 mgL^−1^ Kin**	0.50 ± 0.577 d	0.50 ± 0.577 e	0.100 ± 0.115 hi	0.090 ± 0.103 d	0.050 ± 0.057 hi
VS + 4 mgL^−1^ Kin	0.00 ± 0.00 e	0.00 ± 0.00 f	0.00 ± 0.00 i	0.00 ± 0.00 i	0.00 ± 0.00 i
SH + Control	0.00 ± 0.00 e	0.00 ± 0.00 f	0.00 ± 0.00 i	0.00 ± 0.00 e	0.00 ± 0.00 i
SH + 1.5 mgL^−1^ BAP	0.00 ± 0.00 e	0.00 ± 0.00 f	0.00 ± 0.00 i	0.00 ± 0.00 e	0.00 ± 0.00 i
SH + 3 mgL^−1^ BAP	0.00 ± 0.00 e	0.00 ± 0.00 f	0.00 ± 0.00 i	0.00 ± 0.00 e	0.00 ± 0.00 i
SH + 4 mgL^−1^ BAP	0.00 ± 0.00 e	0.00 ± 0.00 f	0.00 ± 0.00 i	0.00 ± 0.00 e	0.00 ± 0.00 i
SH + 1.5 mgL^−1^ Kin	0.00 ± 0.00 e	0.00 ± 0.00 f	0.00 ± 0.00 i	0.00 ± 0.00 e	0.00 ± 0.00 i
SH + 3 mgL^−1^ Kin	0.00 ± 0.00 e	0.00 ± 0.00 f	0.00 ± 0.00 i	0.00 ± 0.00 e	0.00 ± 0.00 i
SH + 4 mgL^−1^ Kin	0.00 ± 0.00 e	0.00 ± 0.00 f	0.00 ± 0.00 i	0.00 ± 0.00 e	0.00 ± 0.00 i
B5 + Control	0.00 ± 0.00 e	0.00 ± 0.00 f	0.00 ± 0.00 i	0.00 ± 0.00 e	0.00 ± 0.00 i
B5 + 1.5 mgL^−1^ BAP	0.00 ± 0.00 e	0.00 ± 0.00 f	0.00 ± 0.00 i	0.00 ± 0.00 e	0.00 ± 0.00 i
B5 + 3 mgL^−1^ BAP	0.00 ± 0.00 e	0.00 ± 0.00 f	0.00 ± 0.00 i	0.00 ± 0.00 e	0.00 ± 0.00 i
B5 + 4 mgL^−1^ BAP	0.00 ± 0.00 e	0.00 ± 0.00 f	0.00 ± 0.00 i	0.00 ± 0.00 e	0.00 ± 0.00 i
B5 + 1.5 mgL^−1^ Kin	0.00 ± 0.00 e	0.00 ± 0.00 f	0.00 ± 0.00 i	0.00 ± 0.00 e	0.00 ± 0.00 i
B5 + 3 mgL^−1^ Kin	0.00 ± 0.00 e	0.00 ± 0.00 f	0.00 ± 0.00 i	0.00 ± 0.00 e	0.00 ± 0.00 i
B5 + 4 mgL^−1^ Kin	0.00 ± 0.00 e	0.00 ± 0.00 f	0.00 ± 0.00 i	0.00 ± 0.00 e	0.00 ± 0.00 i

The same lowercase letters indicate no significant differences between treatments according to the LSD test at a 5% probability level (mean ± standard deviation).

## Data Availability

The original contributions presented in the study are included in the article; further inquiries can be directed to the corresponding author.

## Appendix A. The Nutrient Concentrations of the Six Plant Tissue Culture Basal Media Used in the Experiments

**Basal Medium**	**B5**	**MS**	**VS**	**WPM**	**DKW**	**SH**
** Macronutrient components (mgL^−1^)**						
KNO_3_	2500	1900	1900	-	-	
K_2_SO_4_	-	-	-	990	1559	
NH_4_NO_3_	-	1650	1650	400	1416	
Ca(NO_3_)_2_.4H_2_O	-	-	-	556	1948	
NH_4_H_2_PO_4_	-	-	-	-	-	
NaH_2_PO_4_.H_2_O	150	-	-	-	-	
(NH_4_)_2_.SO_4_	134	-	-	-	-	
MgSO_4_.7H_2_O	250	370	370	370	740	
KH_2_PO_4_	-	170	170	170	265	
CaCl_2_.2H_2_O	150	440	440	96	149	
** Micronutrient components (mgL^−1^)**						
H_3_BO_3_	3	6.2	6.2	6.2	48	
KI	0.75	0.83	0.83	-	-	
MnSO_4_.H_2_O	10	16.9	16.9	22.3	33.5	
ZnSO_4_.7H_2_O	2	10.6	10.6	8.6	-	
Zn(NO_3_)_2_.6H_2_O	-	-	-	-	17	
CuSO_4_.5H_2_O	0.039	0.025	0.025	0.25	0.25	
Na_2_MoO_4_.2H_2_O	0.25	0.25	0.25	0.25	0.39	
CoCl_2_.2H_2_O	0.025	0.025	0.025	-	-	
NiSO_4_.6H_2_O	-	-	-	-	0.005	
FeSO_4_.7H_2_O	27.8	27.8	27.8	27.8	33.8	
Na_2_EDTA	37.3	37.3	37.3	37.3	45.4	
** Vitamins and Organics (mgL^−1^)**						
*Myo*-inositol	100	100	100	100	100	
Nicotinic acid	1	0.5	0.5	0.5	1	
Pyridoxine-HCl	1	0.5	0.5	-	0.5	
Thiamine-HCl	10	0.1	0.1	1.6	2	
Glycine	-	2	2	-	20	
L-Glutamine	-	-	-	-	250	
Sucrose (gL^−1^)	30	30	30	30	30	30
pH	5.8	5.8	5.8	5.8	5.8	5.8
